# Mutation Spectrum and De Novo Mutation Analysis in Stickler Syndrome Patients with High Myopia or Retinal Detachment

**DOI:** 10.3390/genes11080882

**Published:** 2020-08-03

**Authors:** Li Huang, Chonglin Chen, Zhirong Wang, Limei Sun, Songshan Li, Ting Zhang, Xiaoling Luo, Xiaoyan Ding

**Affiliations:** State Key Laboratory of Ophthalmology, Zhongshan Ophthalmic Center, Sun Yat-sen University, 54 Xianlie Road, Guangzhou 510060, China; huangli@gzzoc.com (L.H.); Chenchonglin@gzzoc.com (C.C.); wangzhirong@gzzoc.com (Z.W.); Sunlimei@gzzoc.com (L.S.); Lisongshan@gzzoc.com (S.L.); Zhangting@gzzoc.com (T.Z.); Luoxiaoling@gzzoc.com (X.L.)

**Keywords:** Stickler syndrome, high myopia, retinal detachment, de novo mutations, *COL2A1*, *COL11A1*

## Abstract

Stickler syndrome is a connective tissue disorder that affects multiple systems, including the visual system. Seven genes were reported to cause Stickler syndrome in patients with different phenotypes. In this study, we aimed to evaluate the mutation features of the phenotypes of high myopia and retinal detachment. Forty-two probands diagnosed with Stickler syndrome were included. Comprehensive ocular examinations were performed. A targeted gene panel test or whole exome sequencing was used to detect the mutations, and Sanger sequencing was conducted for verification and segregation analysis. Among the 42 probands, 32 (76%) presented with high myopia and 29 (69%), with retinal detachment. Pathogenic mutations were detected in 35 (83%) probands: 27 (64%) probands had *COL2A1* mutations, and eight (19%) probands had *COL11A1* mutations. Truncational mutations in *COL2A1* were present in 21 (78%) probands. Missense mutations in *COL2A1* were present in six probands, five of which presented with retinal detachment. De novo *COL2A1* mutations were detected in 10 (37%) probands, with a mean paternal childbearing age of 29.64 ± 4.97 years old. The mutation features of probands with high myopia or retinal detachment showed that the probands had a high prevalence of *COL2A1* mutations, truncational mutations, and de novo mutations.

## 1. Introduction

Stickler syndrome is a connective tissue disorder that was first reported by Stickler et al. in 1965 [1]. The characteristics of Stickler syndrome include ocular findings of myopia, cataracts, and retinal detachment; hearing loss that is both conductive and sensorineural; midfacial underdevelopment and cleft palate (either alone or as part of the Robin sequence); and mild spondyloepiphyseal dysplasia and/or precocious arthritis [2]. The estimated prevalence of Stickler syndrome among neonates is approximately 1:7500 to 1:9000 [3].

Mutations in the following genes are considered causative of Stickler syndrome: *COL2A1* (OMIM, no. 120140) [4], *COL11A1* (OMIM, no. 120280) [5], *COL11A2* (OMIM, no. 120290) [6], *COL9A1* (OMIM, no. 120210) [7], *COL9A2* (OMIM, no. 120260) [8], *COL9A3* (OMIM, no. 120270) [9], and *LOXL3* (OMIM, no. 607163) [10]. *COL2A1*, *COL11A1*, and *COL11A2* mutations are inherited as autosomal dominant traits, whereas *COL9A1*, *COL9A2*, *COL9A3*, and *LOXL3* mutations are inherited as recessive traits [2].

Ocular involvement is the main indication for the diagnosis of Stickler syndrome [2,11]. In patients with *COL2A1* mutations, myopia is the most common sign, which is present in 90% of patients, followed by vitreous changes, detected in 40% of patients, and cataracts, in 30% of patients. Retinal abnormalities, such as lattice degeneration, retinal holes, retinal detachment, or retinal tears, are present in nearly 20% of patients [12].

In this study, we recruited Stickler syndrome patients with initial symptoms of high myopia or retinal detachment to evaluate the molecular genetic spectrum and the relationship between the genetic findings and the phenotypes of high myopia and retinal detachment.

## 2. Materials and Methods 

A total of 84 eyes of 42 probands from 42 unrelated families with Stickler syndrome were enrolled in our study. This study was performed in accordance with the guidelines of the Declaration of Helsinki and was approved by the Institutional Review Board of the Zhongshan Ophthalmic Center (approval code 2014MEKY048). Informed consent was obtained from all participants or their guardians prior to the collection of the clinical data and genomic samples. 

Genomic DNA was extracted from the leukocytes of venous blood using previously reported methods [13]. Previously published diagnostic criteria for Stickler syndrome [11]—based on assigning points for clinical features, family history data, and molecular data [2]—were used as follows: orofacial abnormalities, including cleft palate (2 points) and characteristic facial features (1 point); characteristic vitreous changes or retinal abnormalities (2 points); auditory abnormalities, including high-frequency sensorineural hearing loss (2 points) and hypermobile tympanic membranes (1 point); skeletal abnormality (1 point); and family history or molecular data (1 point). If the score was ≥5 points, a diagnosis of Stickler syndrome was established. For the patients with a score <5 but vitreous abnormalities and characteristic facial features making them highly suspect for Stickler syndrome, we will consider the following criteria presented by Snead et al. [11]: (1) a congenital vitreous anomaly and, in addition, any three of the following: (2) myopia with onset before 6 years of age, usually stable; (3) retinal detachment or para-vascular pigmented lattice degeneration; (4) joint hypermobility with an abnormal Beighton score, with or without radiological evidence of joint degeneration; (5) audiometric confirmation of a sensorineural hearing defect; and (6) midline clefting. 

Targeted gene panel (TGP) sequencing was performed in 24 patients who visited us from 1 January 2015 to 31 December 2017, and whole-exome sequencing (WES) was performed in 18 patients from 1 January 2018. Sanger sequencing was used to validate the detected variants, and co-segregation analysis was performed in available family members. The TGP contained 386 genes causing inherited retinal disease referred to the retinal information network (RetNet, https://sph.uth.edu/retnet/), including five genes (*COL2A1*, *COL11A1*, *COL11A2*, *COL9A1*, and *COL9A2*) causing Stickler syndrome. The WES and TGP sequencing were performed using the Illumina MiSeq platform (Illumina, Madison, WI, USA) with a SeqCap EZ Library SR kit (Roche NimbleGen Roche, Inc., Madison, WI, USA), a MiSeq kit v2, and a PhiX Control kit v3. The average sequencing depth was set to 100-fold. The Strand Next Generation Sequence (NGS) software version 2.0 (Strand Scientific Intelligence Inc, Miami, FL, USA) was used to align the sequencing reads to the hg19 assembly in the Genome Browser of the University of California, Santa Cruz Genomics Institute. Variation annotation was conducted using single nucleotide polymorphism (SNP) effects analysis software. SIFT (http://sift.jcvi.org/www/SIFT_enst_submit.html), Polyphen2 (http://genetics.bwh.harvard.edu/pph2/), LRT (http://www.genetics.wustl.edu/jflab/lrt_query.html), MutationTaster (http://mutationtaster.org), MutationAssessor (http://mutationassessor.org/r3/), FATHMM (http://fathmm.biocompute.org.uk), MetaSVM (www.openbioinformatics.org/annovar), and MetaLR (www.openbioinformatics.org/annovar) were used for the bioinformatics analysis. All the mutation nomenclature followed the Human Genome Variation Society guidelines for mutation descriptions (http://www.hgvs.org). The allele frequencies of the variants detected in this study were confirmed using the Exome Aggregation Consortium (ExAC; http://exac.broadinstitute.org).

All carriers underwent a complete ophthalmic investigation, including best corrected visual acuity (BCVA) tests, intraocular pressure measurements, slit lamp examinations of the anterior segment (cornea, iris, and lens), and funduscopic examination. The retinas of the affected eyes were assessed under mydriatic conditions with binocular indirect ophthalmoscopy. Ophthalmic findings at presentation included the lens and vitreous status, type and location of retinal holes, status of the macula, and extent of retinal detachment, which were recorded. Refractive errors were measured by subjective refraction combined with objective refraction. The axial length was measured with an IOLMaster (Zeiss, Berlin, Germany). An ultrasound A system (Compact Ttouch 4.0, Quantel Medical, Cournon-d’Auvergne, France) was used for young children who could not cooperate with the IOLMaster examination and patients with retinal detachment. For fundus examinations, a fundus camera (Zeiss, Oberkochen, Germany), a scanning laser ophthalmoscope (SLO, Opto Inc., Marlborough, MA, USA), or a RetCam (Natus Medical, Inc., Pleasanton, CA, USA) was used, depending on the patient’s age and cooperation ability. High myopia was defined as an axial length greater than 26 mm or a spherical equivalent less than −6.00 D in at least one eye.

## 3. Results

### 3.1. Demographic and Clinical Characteristics

A total of 84 eyes of 42 probands, 23 (55%) male and 19 (45%) female, from 42 unrelated families were included in this study. The Stickler score for each proband is listed in Supplementary Table S1. The diagnosis of Stickler syndrome was based on the sores and especially based on the typical vitreous changes and retinal abnormalities (Supplementary Figure S1). The median age at the first-time examination was eight years old. The P25 and P75 were 5 and 10.25 years, respectively. The median axial length was 27.72 mm, the P25 being 24.15 mm and the P75 being 29.04 mm. The mean spherical equivalent was −11.91 ± 5.37 D.

**Figure 1:**
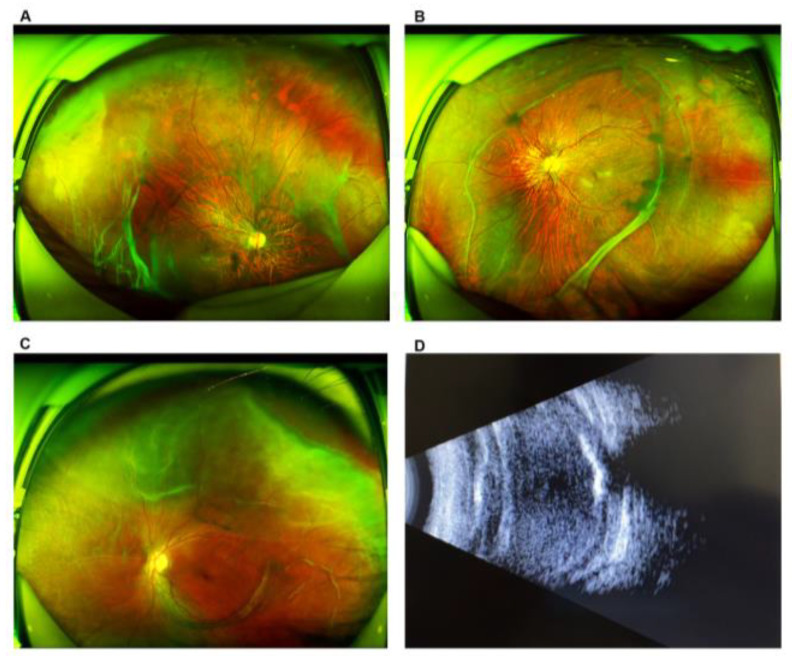
Scanning laser ophthalmoscopy (SLO) and B-scans of patients with Stickler syndrome. (**A**) and (**B**) are from the same proband. (**A**) Vitreous degeneration, multiple retinal holes, and retinal detachment in the right eye. (**B**) Vitreous degeneration and retinal holes in the left eye. (**C**,**D**) are from the same proband. (**C**) Retinal detachment of the left eye. (**D**) B-scan showing retinal detachment, vitreous opacity, and ophthalmatrophia.

Twenty-seven probands had *COL2A1* mutations, eight had *COL11A1* mutations, and seven had no detected mutations. The first examination age was statistically different among the three groups (*p* = 0.048). There was no statistical difference (Kruskal–Wallis test. *p* = 0.260) for the onset age and retinal detachment amongst the different groups of patients. Thirty-two (32/42, 76%) probands had high myopia in total, 21 (21/27, 78%) of whom had *COL2A1* mutations, and 5 (5/8, 63%) had *COL11A1* mutations. Retinal detachment was present in 29 (29/42, 69%) probands, 20 (20/27, 74%) of whom had *COL2A1* mutations, and 4 (4/8, 50%) had *COL11A1* mutations (Table 1, Figure 1). No correlation was found between the Stickler clinical score and retinal detachment (logistic regression, *B* = −0.294, *p* = 0.507). Glaucoma was present in five (5/42, 12%) probands, all of whom had *COL2A1* mutations. Cataracts were present in 10 (10/42, 24%) probands, 6 (6/27, 22%) of whom had *COL2A1*, and 2 (2/8, 25%) had *COL11A1* mutations (Table 1). Other clinical manifestations were a midface and depressed nasal bridge (six probands), mandibular developmental delay (five probands), and cleft palate (five probands).

### 3.2. Genetic Analysis

**Figure 2:**
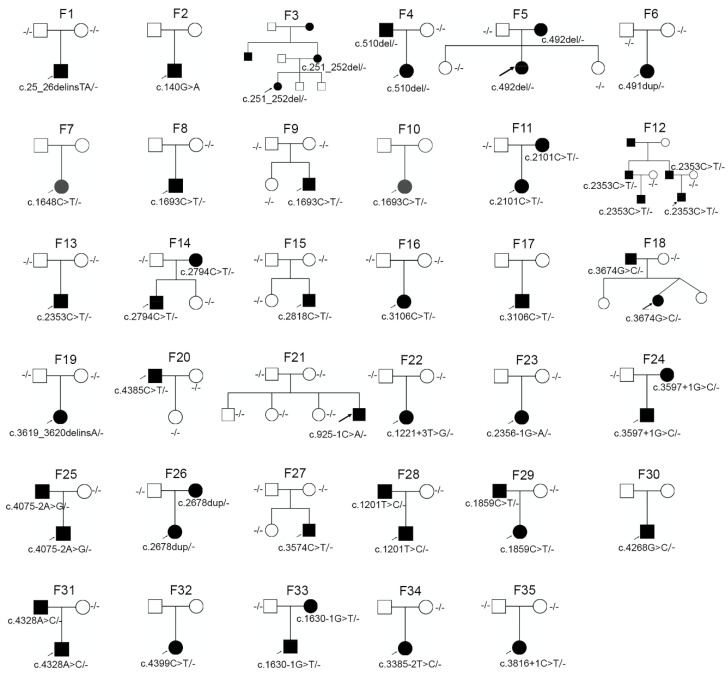
Pedigrees of families with *COL2A1* and *COL11A1* mutations. F1 to F27 are families with *COL2A1* mutations, and F28 to F35 are families with *COL11A1* mutations. The arrows indicate probands; the squares indicate men; the circles indicate women; filled symbols indicate affected individuals; open symbols indicate unaffected individuals.

**Figure 3:**
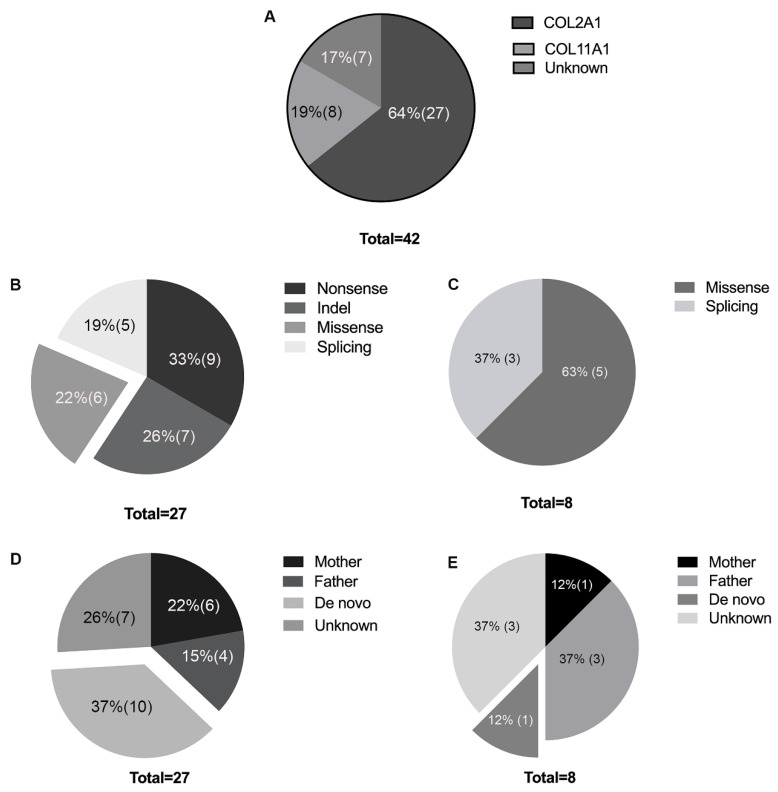
(**A**) Mutation spectrum of the study cohort. (**B**) Composition of *COL2A1* mutation types. (**C**) Composition of *COL11A1* mutation types. (**D**) Mutation inheritance of the *COL2A1* gene. (**E**) Mutation inheritance of *COL11A1*.

**Figure 4:**
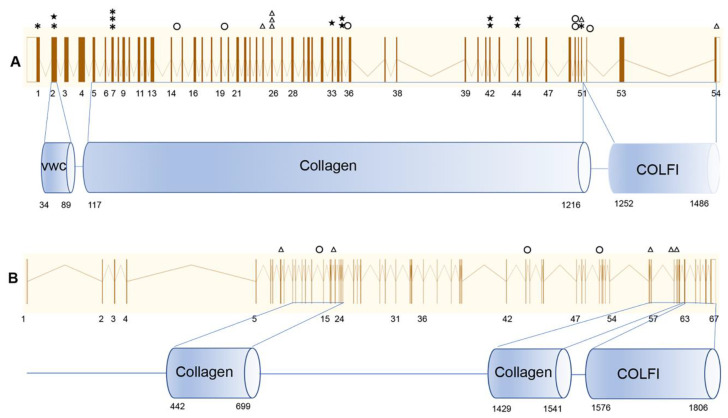
Distribution of *COL2A1* and *COL11A1* mutations. The circles indicate splicing mutations; the triangles indicate missense mutations; the asterisks indicate indel mutations; the pentagrams indicate nonsense mutations. (**A**) Mutations in *COL2A1*. The upper line shows the exons of *COL2A1*, and the lower line shows the domains of the *COL2A1*-encoded protein. (**B**). Mutations in *COL11A1*. The upper line shows the exons of *COL11A1*, and the lower line shows the domains of the *COL2A1* encoded protein.

After TGP sequencing or WES, pathogenic mutations were detected in 35 families (83%), including 27 (64%) with *COL2A1* and 8 (19%) with *COL11A1* mutations (Table 2, Figure 2 and Figure 3A), all in the heterozygous state (Table 2, Figure 2 and Figure 3A). A total of 23 mutations, 11 known mutations [12,14,15,16,17,18,19,20,21] and 12 novel mutations, were detected in *COL2A1*. These mutations were distributed among the exons with no mutation hot spots (Figure 4). Nonsense, indel, splicing, and missense mutations were detected in nine, seven, five, and six families, respectively. Of those, 21 (78%) had truncational mutations (Figure 3B). Eight mutations, two known mutations [18,22] and six novel mutations, were detected in *COL11A1*. Missense and splicing mutations were detected in five (63%) and three (37%) families, respectively (Figure 3C).

### 3.3. De Novo Mutations in COL2A1 and COL11A1 

Segregation analysis in the available family members revealed de novo mutations in 37% (10/27) of the families with *COL2A1* mutations (Figure 3D) and 12% (one in eight) of the families with *COL11A1* mutations (Figure 3E). The family histories and family trees of these families were double-checked to verify the biological parenthood. The reproductive ages of the families with de novo mutations are displayed in Table 3. The mean paternal childbearing age was 29.64 ± 4.97 years, and the mean maternal childbearing age was 27.36 ± 4.97 years (Table 3). Except for two parents with mild myopia, all the other parents of the probands with de novo mutations had normal fundi and a BCVA better than 1.0.

### 3.4. Clinical Manifestation in Probands with COL2A1 Missense Mutations

Four different missense mutations (two known and two novel) were detected in six probands in *COL2A1*. Proband 20, with c.4385C > T (p.Arg1459Cys), developed retinal detachment in the right eye at the age of 19, following which they underwent pars plana vitrectomy (PPV), and glaucoma in their left eye at the age of 30. Proband 18, with c.3674G > C (p.Gly1225Ala), presented with bilateral leukocoria at two months of age and was subsequently diagnosed with retinal detachment bilaterally. Proband 7, with c.1648C > T (p.Arg550Cys), had high myopia and vitreous degeneration with no detected complications at the age of six. Three probands with c.1693C > T (p.Arg565Cys) mutations developed retinal detachment during early childhood bilaterally at 6, 8, and 10 years of age. One of them underwent timely PPV operations, and the other two developed ophthalmatrophia in one eye. In total, there were five out of six probands with missense *COL2A1* mutations that had retinal detachment, whereas 15 out of 21 probands with truncated *COL2A1* mutations had retinal detachment. There was no significant difference (Fisher’s exact test, *p* = 0.498) in the numbers with retinal detachment between the probands with missense and truncated COL2A1 mutations.

## 4. Discussion

In this study, we evaluated the mutation spectrum of patients with Stickler syndrome whose initial symptom was high myopia or retinal detachment.

To date, mutations in seven genes (*COL2A1* [4], *COL11A1* [5], *COL11A2* [6], *COL9A1* [7], *COL9A2* [8], *COL9A3* [9], and *LOXL3* [10]) have been reported to cause Stickler syndrome. *COL2A1* mutations explained the majority (80–90%) of cases [18,23], followed by *COL11A1* mutations (10–20%) [23]. *COL11A2*, *COL9A1*, *COL9A2*, *COL9A3*, and *LOXL3* explained only a few cases, mostly documented in case reports or case series [8,10,24,25,26]. These observations are consistent with this study’s findings. We detected mutations in only *COL2A1* (64%) and *COL11A2* (19%) and no mutations in the other genes. There may be several reasons for this. First, this study included patients with initial signs of high myopia or retinal detachment; however, *COL11A2* mutations have been reported in non-ocular Stickler syndrome [25,27]. Second, patients with *COL9A1* and *COL9A3* mutations mainly present with moderate to severe myopia and rarely with retinal detachment [24,28]. Finally, the rest of the genes are inherited as autosomal recessive traits, whereas the families recruited in this study were autosomal dominant or sporadic cases. In this study, the first examination age was statistically significantly different among the three groups (*p* = 0.048). However, no statistically significant difference was found in the patient numbers with retinal detachment among the three groups. Perhaps longer periods of follow up and larger numbers of patients would provide additional insights into the different clinical trajectories and outcomes of *COL2A1* and *COL11A1* mutant patients.

Various types of mutations in *COL2A1* have been reported, and truncation mutations (nonsense, frameshift, or splicing) that result in a premature stop codon are present in most patients with Stickler syndrome [1,16]. This is in line with the findings in our study, where truncation mutations were detected in 78% of the probands with *COL2A1* mutations. We also detected four missense mutations in six probands, five of whom presented with retinal detachment, which is a fairly severe ocular complication.

De novo mutations in Stickler syndrome have only been reported in a few cases [29]. The proportion of cases caused by de novo pathogenic variants is unknown. In this study, we observed a large proportion of probands with de novo mutations (37% *COL2A1* and 12% *COL11A1*) because these mutations could not be detected in the leukocyte DNA of either parent. The actual proportion of probands with de novo mutations may have been higher as 10 probands were of unknown inheritance. De novo mutations have been established as the cause of early-onset disorders ranging from rare congenital diseases to common neurodevelopment disorders [30,31,32]. Studies have demonstrated that the majority of germline de novo mutations have a paternal origin and that a higher paternal age and the number of de novo mutation increases with paternal childbearing age [33,34]. In this study, we evaluated both the paternal and maternal ages. The paternal childbearing age (29.64 ± 4.97 years old) might be one of the reasons for the high proportion of de novo mutations in this study. Further studies are needed to ascertain the origins of the mutations.

This study was a real-world single-center study. Its sample size was limited due to the rarity of Stickler syndrome and all patients being recruited in a single department (the Pediatric Ophthalmology Department of Zhongshan Ophthalmic Center). The participants presented with an initial sign of high myopia or retinal detachment, which means that the identified mutation spectrum can only be considered representative of the specific phenotypes of Stickler syndrome patients. In this study, we did not use the genomic markers to verify parentage, and the parentage of families with de novo mutations will be further confirmed by genomic markers.

## 5. Conclusions

We investigated the mutation features in Stickler syndrome patients with high myopia or retinal detachment. Mutations were detected only in *COL2A1* (64%) and *COL11A1* (19%). No mutations were detected in other genes known to be associated with Stickler syndrome. De novo mutations accounted for 37% of all *COL2A1* mutations.

## Figures and Tables

**Table 1:** Demographic and clinical data for this study.

Variable	Total	*COL2A1*	*COL11A1*	Unknown
Number (total)	42	27 (64%)	8 (19%)	7 (21%)
Male (n, %)	23 (55%)	14 (52%)	4 (50%)	5 (71%)
Female (n, %)	19 (45%)	13 (48%)	4 (50%)	2 (29%)
Age (years) (median (P25, P75))	8 (5, 10.25)	7 (5, 11)	10 (5.75, 20.25)	12 (4, 18)
AL (mm) (median (P25, P75))	27.20 (24.15, 29.04)	27.06 (24.99, 28.59)	24.96 (23.58, 29.16)	28.71 (21.30, 29.83)
SE (D) (Mean ± SD)	−11.91 ± 5.37	−11.64 ± 4.95	−9.43 ± 7.27	−15.58 ± 3.74
HM (n, %)	32 (76%)	21 (78%)	5 (63%)	6 (86%)
RD (n, %)	29 (69%)	20 (74%)	4 (50%)	5 (71%)
RD (eyes, %)	37 (44%)	25 (46%)	5 (31%)	7 (50%)
Glaucoma (n, %)	5 (12%)	5 (19%)	0	0
Cataract (n, %)	10 (24%)	6 (22%)	2 (25%)	2 (29%)

Note: Age, Age at first examination; HM, High myopia; AL, Axial length; SE, Spherical equivalent; RD, Retinal detachment.

**Table 2:** Mutations detected in this study.

Family ID	Sequence	Gene	Exon	Mutation Type	Location	DNA Change	Amino Acid Change	ExAC—All	ExAC—East Asian	Reference
1	TGP	*COL2A1*	1	indel	chr12: 48398079	c.25_26delinsTA	p.Thr9*	0	0	novel
2	TGP	*COL2A1*	2	nonsense	chr12:48393854	c.140G > A	p.Trp47*	0	0	novel
3	TGP	*COL2A1*	2	indel	chr12:48393736	c.251_252del	p.Glu84Valfs*8	0	0	novel
4	TGP	*COL2A1*	7	indel	chr12:48391409	c.510del	p.Gly171Valfs*28	0	0	[21]
5	WES	*COL2A1*	7	indel	chr12:48393736	c.492del	p.Gly165Valfs*34	0	0	novel
6	TGP	*COL2A1*	7	indel	chr12:48391428	c.491dup	p.Gly165Trpfs*24	0	0	novel
7	TGP	*COL2A1*	25	missense	chr12:48379543	c.1648C > T	p.Arg550Cys	0	0	[14]
8	WES	*COL2A1*	26	missense	chr12:48379358	c.1693C > T	p.Arg565Cys	0	0	[15]
9	TGP	*COL2A1*	26	missense	chr12:48379358	c.1693C > T	p.Arg565Cys	0	0	[15]
10	TGP	*COL2A1*	26	missense	chr12:48379358	c.1693C > T	p.Arg565Cys	0	0	[15]
11	WES	*COL2A1*	33	nonsense	chr12:48376723	c.2101C > T	p.Arg701*	0	0	[16]
12	TGP	*COL2A1*	35	nonsense	chr12:48375892	c.2353C > T	p.Arg785*	0	0	[21]
13	WES	*COL2A1*	35	nonsense	chr12:48375892	c.2353C > T	p.Arg785*	0	0	[21]
14	TGP	*COL2A1*	42	nonsense	chr12:48372481	c.2794C > T	p.Arg932*	0	0	[15]
15	WES	*COL2A1*	42	nonsense	chr12:48372457	c.2818C > T	p.Arg940*	0	0	[19]
16	TGP	*COL2A1*	44	nonsense	chr12:48371798	c.3106C > T	p.Arg1036*	1/114802	0/8542	[17]
17	TGP	*COL2A1*	44	nonsense	chr12:48371798	c.3106C > T	p.Arg1036*	1/114802	0/8542	[17]
18	TGP	*COL2A1*	51	missense	chr12:48369312	c.3674G > C	p.Gly1225Ala	0	0	novel
19	WES	*COL2A1*	51	indel	chr12:48369362	c.3619_3620delinsA	p.Pro1207Thrfs*20	0	0	novel
20	TGP	*COL2A1*	54	missense	chr12:48367279	c.4385C > T	p.Arg1459Cys	3/121410	0/8654	novel
21	WES	*COL2A1*	Intro 14	splicing	chr12:48387286	c.925-1C > A	_	0	0	[18]
22	WES	*COL2A1*	Intro 19	splicing	chr12:48381391	c.1221+3T > G	_	0	0	novel
23	TGP	*COL2A1*	Intro 35	splicing	chr12:48375613	c.2356-1G > A		0	0	novel
24	TGP	*COL2A1*	Intro 50	splicing	chr12:48369745	c.3597+1G > C	_	0	0	novel
25	WES	*COL2A1*	Intro 52	splicing	chr12:48368116	c.4075-2A > G		0	0	Novel
26	WES	*COL2A1*	40	indel	chr12:48373792	c.2678dup	p.Pro893fs	0	0	[12]
27	WES	*COL2A1*	50	nonsense	chr12:48369769	c.3574C > T	p.Arg1192*	0	0	[20]
28	TGP	*COL11A1*	8	missense	chr1:103488342	c.1201T > C	p.Phe401Leu	1/119952	1/8594	novel
29	WES	*COL11A1*	19	missense	chr1:103470204	c.1859C > T	p.Pro620Leu	0	0	novel
30	WES	*COL11A1*	57	missense	chr1:103363724	c.4268G > C	p.Gly1423Ala	0	0	novel
31	WES	*COL11A1*	58	missense	chr1:103356035	c.4328A > C	p.Lys1443Thr	4/102722	2/7546	novel
32	TGP	*COL11A1*	59	missense	chr1:103355112	c.4399C > T	p.Gln1467*	2/120730	2/8600	novel
33	WES	*COL11A1*	intro 14	splicing	chr1:103474073	c.1630-1G > T		0	0	novel
34	TGP	*COL11A1*	intro 43	splicing	chr1:103404646	c.3385-2T > C	_	0	0	[18]
35	TGP	*COL11A1*	intro 50	splicing	chr1:103381186	c.3816+1C > T	_	0	0	[22]

Note: TGP, Targeted gene pane; WES, Whole exome sequencing. *, stop codon.

**Table 3:** Childbearing ages of the parents whose children had de novo mutations.

Family ID	Gene	DNA Changes	Amino Acid Changes	Paternal Childbearing Age (Years)	Maternal Childbearing Age (Years)
1	*COL2A1*	c.25_26delinsTA	p.Thr9*	30	31
6	*COL2A1*	c.491dup	p.Gly165Trpfs*24	30	30
9	*COL2A1*	c.1693C > T	p.Arg565Cys	29	21
13	*COL2A1*	c.2353C > T	p.Arg785*	24	23
15	*COL2A1*	c.2818C > T	p.Arg940*	34	33
16	*COL2A1*	c.3106C > T	p.Arg1036*	26	23
19	*COL2A1*	c.3619_3620delinsA	p.Pro1207Thrfs*20	33	33
21	*COL2A1*	c.925-1C > A	_	38	28
22	*COL2A1*	c.1221 + 3T > G	_	34	30
27	*COL2A1*	c.3574C > T	p.R1192*	21	19
35	*COL11A1*	c.3816 + 1C > T	_	27	30
Mean ± SD				29.64 ± 4.97	27.36 ± 4.97

Note: *, stop codon.

## Supplementary Materials

The following are available online at https://www.mdpi.com/2073-4425/11/8/882/s1. Figure S1: Vitreous changes or retinal abnormalities from Scanning laser ophthalmoscopy (SLO), Table S1: Diagnose of the probands.
