# Supplementary material for: Mutation Spectrum and De Novo Mutation Analysis in Stickler Syndrome Patients with High Myopia or Retinal Detachment
Source: Genes (Basel). 2020 Aug 3;11(8):882. doi: 10.3390/genes11080882 (PMC7464315; doi:10.3390/genes11080882)
Supplement: Supplementary file 1 [file genes-11-00882-s001.zip › Supplementary Figure 1-20200706.docx]

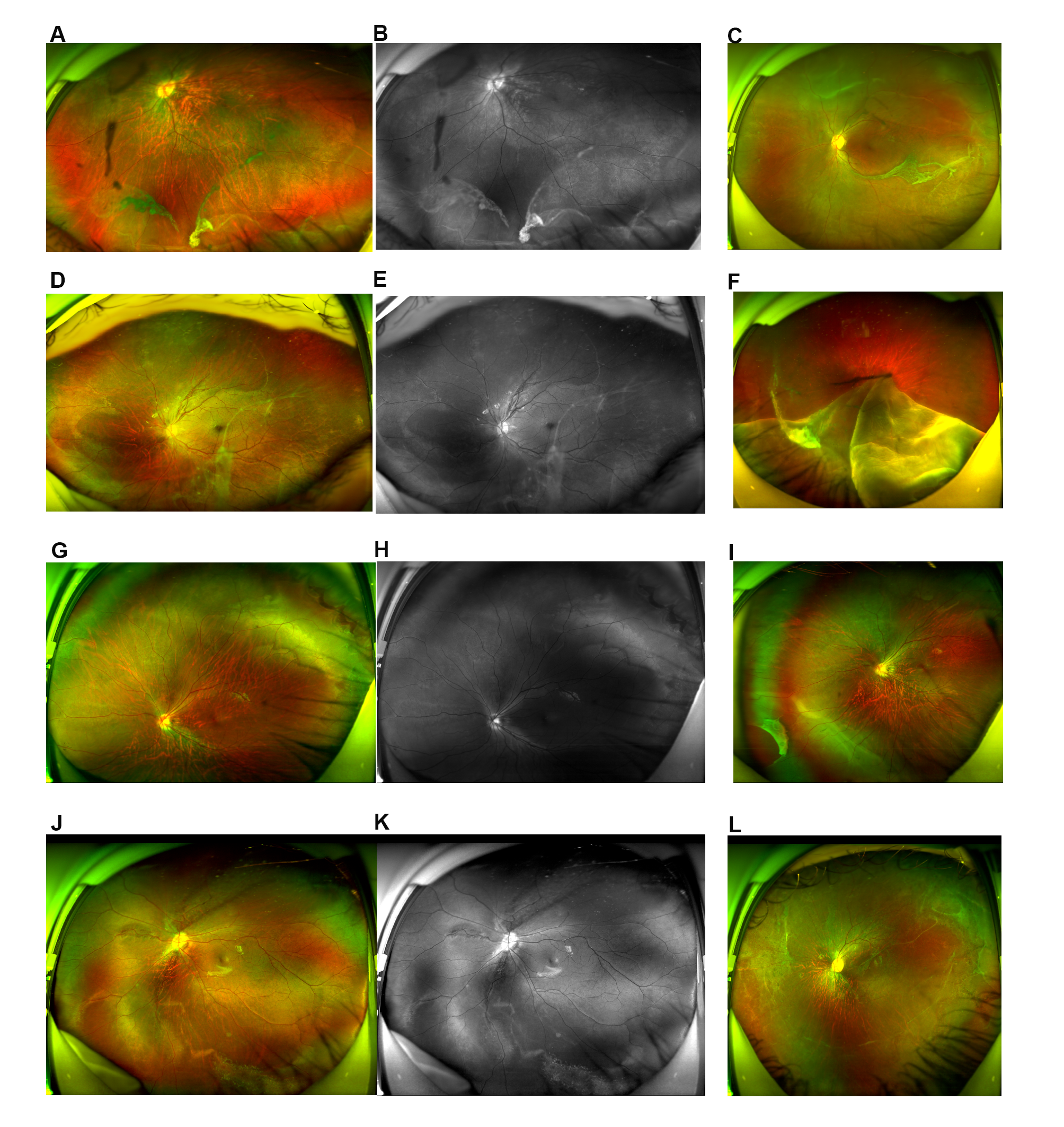


**Supplementary Figure 1.** Vitreous changes or retinal abnormalities from Scanning laser ophthalmoscopy (SLO). A and B, typical membranous vitreous change from proband 20. C. typical membranous vitreous change from proband 11. D and E, beaded vitreous change from proband 18. F. retinal detachment from proband 14. G and H, typical dialysis of ora serrata from proband 25. I, retinal tear combined with retinal detachment from proband 25. J and K, para-vascular pigmented lattice degeneration from proband 24. L. membranous vitreous change and peripheral retinal degeneration from proband 23.
